# Impact of non-coding RNAs on resistance to imatinib in chronic myelogenous leukemia

**DOI:** 10.1016/j.lrr.2025.100529

**Published:** 2025-07-10

**Authors:** Fatemeh Ensafi Talemi, Soudeh Ghafouri-Fard

**Affiliations:** a Student Research Committee, School of Medicine, Shahid Beheshti University of Medical Sciences, Tehran, Iran; b Department of Medical Genetics, Shahid Beheshti University of Medical Sciences, Tehran, Iran

**Keywords:** lncRNA, miRNA, circRNA, Imatinib, Chronic myelogenous leukemia

## Abstract

Imatinib is approved as the first-line treatment for newly diagnosed chronic myelogenous leukemia (CML). In spite of profound response in the majority of patients, resistance occurs in a subgroup of CML cases. Recently, it has been demonstrated that different classes of non-coding RNAs can modulate response to this tyrosine kinase inhibitor. Recognition of the role of these transcripts in this process not only expands our knowledge about the molecular mechanisms of imatinib resistance, but also provides novel strategies for combating this phenotype. The current review summarizes the role of non-coding RNAs in this process and suggests novel candidates for further studies in this field to enhance therapeutic response to imatinib.

## 1 Introduction

Chronic myelogenous leukemia (CML) is a malignancy that mainly affects adults. In 2024, the estimated new cases and estimated deaths from CML in the United States are 9280 and 1280, respectively [1]. This malignancy is among those experiencing significant gains in the survival rate, possibly due to improved treatment protocols, particularly the advent of targeted therapies. To be specific, the 5-year relative survival rate for CML has been increased more than three times, from the mid-1970s to 2019, with tyrosine kinase inhibitors (TKIs) offering near-normal life expectancy for the majority of patients [2].

Being approved as the first-line treatment for newly diagnosed CML, imatinib represents one of the most successful translations of molecular sciences into the clinical setting. In fact, it was the first signal transduction inhibitor used for management of patients [3]. It precludes the function of BCR-ABL1 protein in the oncogenic pathway through binding with its kinase domain [4]. Thus, it blocks the transmission of proliferative signals to the nucleus and induces leukemic cell apoptosis [4].

Although this agent induces long-lasting and successful responses in most patients, resistance to imatinib remains a major clinical challenge [5]. In spite of development of three generations of TKIs, drug resistance and progression to acute phase happens in 5–10 % of CML patients [6]. By definition, imatinib resistance occurs in three distinct situations, namely failure to achieve complete hematologic response and BCR-ABL1 transcript levels ≤ 10 % after 3–6 months of therapy; failure to achieve complete cytogenetic response or BCR-ABL1 transcripts ≤ 1 % after 1 year; or cytogenetic or hematologic relapse at any time after 1 year of imatinib therapy. The latter is considered as secondary resistance [7]. Amplification of *BCR*-*ABL1* gene and mutations in the catalytic domain of the encoded protein are two main mechanisms that have been identified for resistance phenotype for more than two decades [8]. However, recent attempts in the characterization of molecular mechanisms of imatinib resistance have focused on the role of non-coding fraction of the genome as one of the most important regulatory mechanisms. The current review summarizes the impact of long non-coding RNAs (lncRNAs), microRNAs (miRNAs) and circular RNAs (circRNAs) in imatinib resistance in CML.

### 1.1 LncRNAs and imatinib resistance

Among lncRNAs that affect response to imatinib is the oncogenic lncRNA CCAT2. This lncRNA has been found to be up-regulated in newly diagnosed CML patients during the chronic phase compared with healthy controls. In addition, CCAT2 levels have been associated with therapy response at 3 and 6 months. Besides, CCAT2 levels have been significantly associated with both spleen size and EUTOS sore. Finally, over-expression of CCAT2 at diagnosis has been linked to imatinib resistance [9].

SNHG5 is another lncRNA that affect response to imatinib. This lncRNA exerts its role through sponging miR-205-5p and releasing ABCC2 from inhibitory effects of this miRNA. Since ABCC2 encodes a multidrug resistance (MDR) protein participating in the chemoresistance, it is not surprising that SNHG5/miR-205-5p/ABCC2 is a functional axis in induction of resistance to this drug in CML. The functional links between these molecules have been verified by luciferase reporter assay and RNA immune-precipitation. CML patients have been shown to have elevated levels of both SNHG5 and ABCC2 in their peripheral blood cells of the CML patients compared with healthy controls [10].

Similarly, UCA1 has been identified as an essential modulator of MDR1 through experiments in a model system of leukemia cell lines with a gradual increase of MDR1 expression and imatinib resistance. Up-regulation of UCA1 has led to over-expression of MDR1 and imatinib resistance in CML cells. These effects are mainly exerted through binding of this lncRNA with miR-16 [11].

MEG3 is another lncRNA that affect expression of a certain miRNA and its associated MDRs. However, contrary to SNHG5, it induces sensitivity to imatinib. Expression assays have shown down-regulation of MEG3 imatinib-resistant CML patients and imatinib-resistant K562 cells. Forced up-regulation of MEG3 in imatinib-resistant K562 cells has evidently reduced cell proliferation, enhanced cell apoptosis, upturned imatinib resistance, and decreased MRP1, MDR1, and ABCG2 levels. Functional studies have suggested that MEG3 regulates response to imatinib possibly through regulating miR-21 [12].

IRAIN is another lncRNA with constitutive low expression in CML. This BCR-ABL1-independent 1ncRNA has a role in imatinib resistance. Its silencing has reduced the sensitivity of CD34+ CML blasts and cell lines to imatinib, while its up-regulation has induced drug sensitivity. From a mechanistical point of view, this lncRNA decreases expression of CD44, a membrane receptor that contributes to TKI resistance, through binding to the NF-ƙB subunit p65 to decrease levels of p65 and phosphorylated p65. Notably, combination of imatinib with the demethylating agent decitabine, which increases IRAIN levels, has been suggested as a dual treatment strategy to overcome resistance to TKIs [13].

MALAT1 is another up-regulated lncRNA in CML cells. MALAT1 knock-down has blocked proliferation of CML cells, arrested their cell cycle and improved imatinib sensitivity through targeting miR-328 [14].

LncRNA-IUR1 is another lncRNA with critical role in negative regulation of BCR-ABL1-induced tumorigenesis. This lncRNA is expressed in a very low level in BCR-ABL1-positive cells from CML patients. However, its expression has been induced in ABL-positive leukemic cells after treatment with imatinib. Silencing of lncRNA-IUR1 has enhanced survival of ABL1-transformed human leukemic cells both *in vitro* and *in vivo*. Subsequent experiments have shown that lncRNA-IUR1 suppresses BCR-ABL1-induced tumorigenesis *via* decreasing STAT5-mediated GATA3 expression [15]. Table 1 shows the impact of lncRNAs on resistance of CML to imatinib.

**Table 1:** Impact of lncRNAs on resistance of CML to imatinib (IM).

lncRNA	Alternation in CML	Cell line	Database	Patient samples	Mice model	Targets	Function	Effects	References
PXN‑AS1	Upregulation	K562/ K562-IR LAMA84/ LAMA84-IR HEK-293T	Targetscan/Tarbase/miRDB/miRWalk	14 Bone marrow samples of CML patients (6 IM-responding and 8 non-responding)	BALB/c nude mice	miR-635/GS/Gln/mTOR pathway	Binds to miR-635, thus regulates GS expression and disrupts the Cyclin D/CDK4-CDK6 complex.	Enhances IM resistance	[16]
OIP5-AS1	Upregulation	K562/ 293T/ K562/G01	GEO/ starBase/LncBase/Targetscan	Bone marrow aspiration specimens of 2 normal individuals and 6 CML patients	–	miR-30e-5p/ATG12 Axis	Regulates the expression of ATG12 through binding miR-30e-5p	Enhances IM resistance	[17]
HULC	Upregulation	K562/ K562-R/HS-5	–	Bone marrow samples from 25 healthy volunteers and 66 patients with CML before and after treatment with IM (31 IM-responding and 35 non-responding)	–	miR-150-5p/MCL1	Regulates the PI3K/AKT pathway via downregulating miR-150-5p	Enhances IM resistance	[18]
	Upregulation	K562/ KG-1/ THP-1	–	–	–	miR-200a-3p /c-Myc/Bcl-2/ PI3K/Akt pathway	Modulates c-Myc and Bcl-2 by sequestering miR-200a-3p and maintains cell survival through suppression of pro-apoptosis factors and enhancement of anti-apoptosis factors.	Enhances IM resistance	[19]
H19	Upregulation	K562/ K562-IMR/LAMA84-IMS/LAMA84- IMR/ HEL 92.1.7	GO/KEGG	Whole blood samples from 10 control subjects, 10 CML patients and 8 BCR-ABL1-neg MPN patients	–	miR-675-5p/ RUNX1 axis	Post-transcriptional regulatory pathway driven by miR-675-5p functionally inhibits expression of wild-type RUNX1	Enhances IM resistance	[20]
LNC000093	Downregulation						Regulates cell viability through competing with H19/miR-675-5p-mediated RUNX1 inhibition		
MALAT1	–	K562/KG-1	–	–	–	miR-328/ PCNA/ c-Myc/ cyclin D1 and C/EBPa	Induces proliferation and promotes cell cycle	Enhances IM resistance	[21]
MEG3	Downregulation	K562/K562R	–	68 blood samples from CML patients (34 IM-responding and 34 non-responding)	–	miR-21	Regulates IM resistance through suppressing miR-21	Enhances IM sensitivity	[12]
UCA1	–	K562/ K562IM-R	–	–	–	miR-16/ MDR1	Functions as ceRNA of MDR1 through completely interacting with miR-16.	Enhances IM resistance	[22]
BGL3	Downregulation	K562/293T/Jurkat/QGY-7703/BEL-7402/ HepG2/Huh7	FINDTAR3	4 Bcr-Abl-positive CML samples derived from patients in chronic phases and	Nude mice	PTEN	Regulates cell survival, via upregulating PTEN	Enhances IM sensitivity	[23]

### 1.2 miRNAs and imatinib resistance

Function of a number of mRNAs in imatinib resistance has been assessed when evaluating the role of lncRNAs that act as molecular sponge for them. A number of additional studies have evaluated miRNAs functions independently from lncRNAs. For instance, Jiang et al. have assessed expression profile of STAT5-related miRNAs in two CML cell lines, including the imatinib-sensitive and imatinib-resistant ones. They have found down-regulation of miR-221 in imatinib-resistant cells and blood samples of patients with treatment failure, compared to corresponding controls. They have also detected inverse correlation between expressions of STAT5 and miR-221 CML cells. Moreover, STAT5 has been validated as a direct target of this miRNA. Cumulatively, miR-221/STAT5 axis has been shown to play crucial role in controlling the sensitivity of CML cells to imatinib through regulating the Bcl2/Bax ratio [24].

A high throughput miRNA proofing in imatinib-treated CML patients, including those with complete cytogenetic response as well as primary resistant patients has led to identification of 19 miRNAs that may predict clinical resistance to imatinib. Among these miRNAs have been hsa-miR-199a, hsa-miR-183 and hsa-miR-29c that target transporters being involved in chemoresistance, namely ABCC5, ABCA1 and ABCB6, respectively [25]. miR-409-5p is another miRNA that is possibly involved in imatinib sensitivity possibly though modulating expression of NUP43. Expression of this miRNA has been found to be decreased in child CML patients and cells. Forced up-regulation of miR-409-5p has remarkably suppressed proliferation and arrested cell cycle in the G0/G1 phase. Following overexpression of miR-409-5p, the anti-proliferative effects of imatinib in K562 and KG-1a cells have been improved [26].

Moreover, miRNA profiling of exosomes isolated from imatinib-sensitive and -resistant cells has revealed that miR-125b-5p and miR-99a-5p are over-expressed in resistant cells, their exosomes, and sensitive cells that are treated with resistant-derived exosomes. Meanwhile, miR-210-3p and miR-193b-3p have been found to be down-regulated in resistant cells and their exosomes. Subsequent *in silico* studies have indicated the possible role of CCR5, GRK2, EDN1, ARRB1, P2RY2, LAMC2, PAK3, PAK4 and GIT2 in exosome-mediated transfer of imatinib resistance. Moreover, mTOR, STAT3, MCL1, LAMC1, and KRAS have been predicted to be correlated with imatinib resistance. Since MDR1 transcript levels have been higher in resistant cells and their exosomes compared to sensitive cells and their exosomes, authors have suggested exosomal transfer of MDR1 mRNA [27]. Table 2 shows the impact of miRNAs on resistance of CML to imatinib.

**Table 2:** Impact of miRNAs on resistance of CML to imatinib (IM).

miRNA	Alternation In CML	Cell line	Database	Patient samples	Mice model	Target	Function	Effects	References
miR–629-5p (extracellular vesicles)	Upregulation	K562/K562-Re	–	11 newly diagnosed chronic phase CML patients and 6 blast crisis patients who developed during IM treatment	–	SENP2/PI3K/AKT/mTOR	Inhibits phosphorylation of the PI3K/AKT/mTOR pathway	Enhances IM resistance	[28]
miR-125b-5p	Upregulation	K562S/K562R (Cells and Exosomes)	KEGG/TarBase	–	–	MDR1/MCL1	Associates with IM resistance.	Enhances IM resistance	[29]
miR-99a-5p	Upregulation								
miR–210–3p	Downregulation								
miR-193b-3p	Downregulation								
miR-203	Downregulation	BaF3-BCR/ABL	–	Bone marrow samples from 8 CML patients and peripheral blood of 5 healthy donors	–	ABL /BCR-ABL	Associates with IM sensitivity.	Enhances IM sensitivity	[30]
miR–495–3p	Downregulation	UT-7/ UT–7–11/KCL22	GSE4170/ TCGA-AML/ miRDB/targetscan/ GO-BP	–	–	MDR1/BCR-ABL1	Negatively regulates IM resistance by suppressing MDR1 expression.	Enhances IM sensitivity	[31]
miR-23a	Downregulation	K562 and the drug-resistant variant K562/G01	–	70 Bone marrow samples of CML patients (25 IM-responding and 45 non-responding)	–	CRYAB	Upregulates CRYAB expression and reduces miR-23a-induced cell apoptosis and IM sensitivity in CML.	Enhances IM resistance	[32]
miR-199a-3p	Downregulation	PBMCs/KCL-22/K562/ KBM5/ Ku812	–	Serum samples of 25 patients diagnosed with CML and 25 healthy volunteers	–	mTOR	Promotes cell apoptosis of CML cells by downregulating mTOR.	Enhances IM sensitivity	[33]
miR-199a/b-5p	–	K562/ K562R KU812/ KU812R	–	–	–	WNT2	Negatively regulates the expression of WNT2 and might enhance IM sensitivity in IM-resistant CML cells via inhibiting autophagy and inducing apoptosis.	Enhances IM sensitivity	[34]
miR-199b	Downregulation	–	–	Peripheral blood and bone marrow specimen from 150 CML patients	–	–	Associates with IM resistance.	Enhances IM resistance	[35]
miR-150	Downregulation	–	–	60 Blood samples of CML patients (48 IM-responding and 12 non-responding)	–	–	Associates with IM sensitivity.	Enhances IM sensitivity	[36]
miR‑33a‑5p	Upregulation	K562/IM resistant KG cell line	TargetScan	–	Female nude mice	HMGA2	Inhibits HMGA2 expression in KG cells	Enhances IM sensitivity	[37]
miR-145a-5p	–	K562/ K562-R	–	Bone marrow aspiration specimens of 20 cases of IM-sensitive and 20 cases of IM-resistant (relapsed)	NOD-SCID mice	USP6/GLS1 signaling	Promotes apoptosis and Associates with IM sensitivity.	Enhances IM sensitivity	[38]
has-miR-26a-5p	Upregulation	–	–	Blood specimens from 58 CML patients (30 IM-responding and 28 non-responding)	–	–	Associates with IM resistance.	Enhances IM resistance	[39]
hsa-miR–182-5p	Upregulation								
miR–379-5p	–	HEK293T/ K562/ KU812/ hBMSCs	GEPIA/ GEO/ miRDB/targetscan	–	NOD/SCID mice	AKR1C3	Suppresses AKR1C3 and resulting IM resistance by MAPK/ERK inactivation	Enhances IM sensitivity	[40]
miR–342-5p	Upregulation	K562/MEG01/KU812	GEO	Peripheral blood specimens from 20 CML patients and 13 healthy donors	C57BL/6JNarl mouse	CCND1	Downregulates CCND1 expression and enhanced imatinib-induced DNA double-strand breaks and apoptosis	Enhances IM sensitivity	[41]
miR-126	Upregulation	–	–	Peripheral blood specimens of 100 clinically confirmed by Bcr/ Abl, CML patients and 100 healthy subjects	–	–	Associates with IM sensitivity.	Enhances IM sensitivity	[42]
miR-122	Upregulation								
miR–486-5p	Upregulation	K562/ K562-R	–	Peripheral blood specimens of 36 newly diagnosed cases of CP-CML patients before and after treatment with IM	–	–	Associates with IM sensitivity.	Enhances IM sensitivity	[43]
miR-146a	Upregulation	–	–	Peripheral blood specimens of 75 patients with CML before and after treatment with IM (32 IM-responding and 43 non-responding) and 58 healthy controls	–	c-IAP-/ MCL-1	May inhibit the BCR–ABL+ cell death increasing the leukemic cell survival.	Enhances IM resistance	[44]
miR-146a	Upregulation	–	–	Peripheral blood specimens of 60 patients with CML before and after treatment with IM (48 IM-responding and 12 non-responding) and 20 healthy controls	–	–	Associates with IM sensitivity.	Enhances IM sensitivity	[45]
miR-146a-5p	Downregulation	–	KEGG	Peripheral blood specimens of 8 patients diagnosed with CML in chronic phase, demonstrated primary resistance to IM and 2 healthy normal controls	–	–	Associates with IM resistance.	Enhances IM resistance	[46]
miR-99b-5p									
miR-151a-5p									
miR-451	Downregulation	K562/ K562-R	–	–	–	–	Associates with IM resistance.	Enhances IM resistance	[47]
miR-144	Downregulation	K562/ K562-R	–	–	–	–	Associates with IM resistance.	Enhances IM resistance	[47]
miR-30a	Downregulation	–	–	80 patients with CML (20 new chronic phase adult CML patients, 30 imatinib responder CML patients, 30 resistant patients) and 20 healthy controls	–	Beclin 1	Inhibits autophagy by downregulating Beclin 1	Enhances IM resistance	[48]
	Downregulation	K562	–	Bone marrow mononuclear cells from CML patients	–	Beclin 1/ATG5	Inhibits autophagy by downregulating Beclin 1 and ATG5	Enhances IM resistance	[49]
miR–202-5p	Upregulation	KCL22/K562/ K562G	TargetScan/ TCGA	30 patients with chronic phase of CML (CML-CP) and 30 healthy donors	–	USP15/Caspase-6 axis	Downregulates USP15 and decreases the apoptosis of CML cells by lowering the level of Caspase-6 protein.	Enhances IM resistance	[50]
miR-202	Downregulation	K562/ KU812	–	Peripheral blood specimens from 15 CML patients and 10 healthy donors	–	HK2	Inhibits glycolysis through targetting HK2.	Enhances IM sensitivity	[51]
miR–142-5p	Downregulation	–	TargetScan/MicroCosm Targets/ DIANA-microT	28 Peripheral blood and 17 bone marrow samples from CML (26 IM-responding and 19 non-responding)	–	ABL1/MCL1/SRI/ cKIT	Associates with IM resistance.	Enhances IM resistance	[52]
miR-365a-3p	Downregulation					cKIT	Associates with IM resistance.		
miR–153–3p	Upregulation	KBM5/K562/KBM5R/K562R	–	The blood samples from 44 CML patients (22 IM-responding and 22 non-responding)	–	Bcl‑2	Targets Bcl-2 to promote the development of IM resistance and attenuate IM-induced apoptosis in CML	Enhances IM sensitivity	[53]
miR-221	Upregulation	K562/ KBM5/ K562/G	–	35 Peripheral blood and 17 bone marrow samples from CML (20 IM-responding and 15 non-responding)	–	STAT5B	Downregulates STAT5 and enhances apoptosis and promote proliferation	Enhances IM sensitivity	[24]
miR-21	Upregulation	K562/K562-RC/K562-RD	–	57 blood samples from CML patients (39 IM-responding and 18 non-responding)	–	–	Associates with IM resistance.	Enhances IM resistance	[54]
	Upregulation	–	–	Peripheral blood specimens of 100 patients with CML before and after treatment with IM and 100 healthy controls	–	–	Associates with IM resistance.	Enhances IM resistance	[55]
miR-451	Downregulation	K562/K562-RC/K562-RD	–	57 blood samples from CML patients (39 IM-responding and 18 non-responding)	–	–	Associates with IM resistance.	Enhances IM resistance	[54]
	Downregulation	–	Targetscan/Microcosm	59 blood samples from CML patients (29 IM-responding and 30 non-responding)	–	MYC	Associates with IM resistance.	Enhances IM resistance	[56]
miR-577	Upregulation	K562/KG-1a Before and after treating with IM for 24h	–	Peripheral blood specimens of 18 patients with CML and 28 healthy controls	–	NUP160	Associates with IM sensitivity.	Enhances IM sensitivity	[57]
miR–409-5p	Upregulation	K562/KG-1a Before and after treating with IM for 24h	–	Peripheral blood specimens of 42 children with CML and 40 healthy controls	–	NUP43	Downregulates NUP43 and enhances the inhibitory effect of IM on cell cycle progression.	Enhances IM sensitivity	[26]
miR-96	Downregulation	K562/ MEG01	–	Bone marrow aspiration specimens of CML-CP (*n* = 38), CML-BC (*n* = 12) and samples from iron-deficiency anemia (IDA) patients and healthy (Control, *n* = 22).	–	BCR-ABL1	Inhibits BCRABL1 protein expression, autophosphorylation (pTyr) and STAT5 and MEK/ERK signaling pathway activity without affecting BCR-ABL1 level	Enhances IM sensitivity	[58]
miR–141-5p	Downregulation	K562/K562-G	miRanda/miRbase Targets	21 newly diagnosed CML cases before and after treatment with IM and 14 healthy controls	BALB/c nude mice	RAB32	Suppresses the expression of RAB32 through binding to RAB32 mRNA 3′UTR.	Enhances IM sensitivity	[59]
miR-328	–	K562/K652G/K562R1/K562R2	–	–	–	ABCG2	Sensitizes resistant cells to imatinib via post-transcriptionally decreasing ABCG2 expression,	Enhances IM sensitivity	[60]
miR-214	Downregulation	K562/ K562R	miRanda/ TargetScan/PiTa/RNAhybrid/PICTA	57 blood samples from CML patients (21 IM-responding and 36 non-responding)	–	ABCB1	Binds with the 3′‑UTR seed region of ABCB1 mRNA	Enhances IM resistance	[61]
miR-365 (exosome)	–	K562 cell/K562-G01	–	–	–		Lowers chemosensitivity and apoptosis in imatinib-sensitive CML cells.	Enhances IM resistance	[62]
miR-29a-3p	Upregulation	K562	TargetScan	10 chronic phase Ph+ CML cases of CML before and after treatment with IM and 4 healthy controls		TET2	Induces TET2 silencing and protects cells from IM-induced apoptosis	Enhances IM resistance	[63]
miR–660-5p	Upregulation	K562	TargetScan	10 chronic phase Ph+ CML cases of CML before and after treatment with IM and 4 healthy controls	–	EPAS1	Protects cells from IM-induced apoptosis through EPAS1 targeting.	Enhances IM resistance	
miR–494–3p	Downregulation	K562	TargetScan	10 chronic phase Ph+ CML cases of CML before and after treatment with IM and 4 healthy controls		c-MYC	Associates with IM sensitivity.	Enhances IM sensitivity	
miR-224	Downregulation	K562/ K562R	Target-Scan/ PicTar/ miRandamirSVR	Bone marrow aspiration specimens of 42 newly diagnosed CML patients (15 IM-responding and 27 non-responding)	nude mice	ST3GAL IV	Regulates proliferation and chemosensitivity of CML leukemia cells probably through targeting ST3GAL IV.	Enhances IM resistance	[64]
miR-let-7i									
miR-101	–	K562	TargetScan/miRNA.org	–	–	Jak2 and MCL-1/ c-Myc and hTERT/ STAT5/Bcl-2/CCND1/ STAT5/ NF-κB-regulated anti-apoptotic genes	Associates with IM sensitivity.	Enhances IM sensitivity	[65]
miR-1301	–	K562/KU812/ HEK293	GEO/ TargetScan	Peripheral blood specimens of 4 patients with CML and 2 healthy controls	–	RanGAP1	Affects BCR-ABL distribution in IM-treated K562 cells by downregulating RanGAP1 expression.	Enhances IM sensitivity	[66]
miR-215 (plasma)	–	–	miRTarBase	Peripheral blood specimens of 52 patients with CML before and after treatment with IM and 28 healthy controls	–	–	Associates with IM sensitivity	Enhances IM sensitivity	[67]
miR–486-5p	Upregulation	HEK293	TargetScan/miRanDa	Human cord blood and CML bone marrow samples	NOD-SCID IL2-rg-null (NSG) mice	FoxO1/PTEN/ ARID4b/ AFF3/ TWF1/ PI3KP	Contributes to survival of BCR-ABL transformed cells after IM treatment and its inhibition enhances the sensitivity of CML progenitors to IM-mediated apoptosis.	Enhances IM sensitivity	[68]
miR‑181a	Downregulation	K562/ 293T-17	–	Peripheral blood specimen of a patient with CML and 3 healthy volunteers	–	–	Inhibits the G1/S phase transition of the cell cycle in K562 cells and promotes the differentiation	Enhances IM sensitivity	[69]
miR-424	Downregulation	K562/ K562R/ Meg-01	TargetScan/miRanda/miRWalk algorithms	Peripheral blood samples from 18 newly diagnosed CML patients before treatment and 10 additional healthy volunteers.	–	BCR–ABL	Acts as a tumor suppressor by downregulating BCR–ABL expression and reduces cell growth	Enhances IM sensitivity	[70]
hsa-miR-2278	Upregulation	K562/K562-IMA-3	miRTar/TargetScan	–	–	AKT2/ STAM2/STAT5A	Inhibits leukemic cell proliferation, induced apoptosis, and regain of chemotherapeutic drug response in CML therapy.	Enhances IM sensitivity	[71]
miR-30e	Downregulation	K562/ HL-60/MEG-01/THP-1	TargetScan/PicTar	Peripheral blood specimens of 18 patients with CML before and after treatment with IM and 10 healthy controls	–	ABL	Acts as a tumor suppressor by downregulating BCR–ABL expression.	Enhances IM sensitivity	[72]
miR-21	–	–	–	Bone marrow samples from treatment-naïve patients in CP CML and from healthy donors		AKT/c-Myc	Combination of miR-21 inhibitor and IM significantly decreases AKT phosphorylation and c-Myc expression.	Enhances IM sensitivity	[73]
miR–493-5p	–	K562 treated with IM	GEO/miRbase/miRanda	–	–	IL8	Inhibits IL8 expression and led imatinib sensitivity.	Enhances IM sensitivity	[74]
miR-26a	Upregulation	–	–	Peripheral blood specimens of 75 patients with CML (32 IM-responding and 43 non-responding) and 58 healthy controls	–	c-IAP-/ MCL-1	Suppresses the BCR–ABL+ cell death	Enhances IM resistance	[44]
miR-29c									
miR-130b									
miR-138	Downregulation	K562/ Ku812/ HL-60/Kasumi-1, THP1/Jurkat/Molt-4/NB4	–	Bone marrow samples from 17 CML patients	–	ABL/ CCND3	Downregulates BCR-ABL and ABL by targeting the coding region of the ABL gene.	Enhances IM sensitivity	[75]
miR-148b	–	–	–	Peripheral blood specimens of 59 patients with CML (16 IM-responding and 33 non-responding) and 15 healthy controls	–	–	Associates with IM resistance.	Enhances IM resistance	[76]
miR-181c	Downregulation		TargetScan/miRanda/miRBase/mirTarget2/ Tarbase/PICTAR	bone marrow core biopsies from 9 CML patients (4 IM-resistant and 5 IM-responder patients)	–	–	Associates with IM resistance.	Enhances IM resistance	[77]

### 1.3 circRNAs and imatinib resistance

The impact of circRNAs on imatinib resistance is being discovered in recent years. Using a circRNA microarray, Zhong et al. have found differentially expressed circRNAs between optimal imatinib responder and non-responder CML patients. Among these circRNAs has been hsa_circ_0058493. Over-expression of hsa_circ_0058493 has been associated with the poor response imatinib. Silencing expression of this circRNA has led to inhibition of establishment of imatinib-resistance in CML cells. Mechanistically, circ_0058493 is predicted to serve as a “sponge” for miR-548b-3p Besides, hsa_circ_0058493 has been found to be enriched in the exosomes originated from imatinib-resistant CML cells [78]. circ_SIRT1 is another up-regulated circRNA in CML whose over-expression is correlated with imatinib resistance. Silencing of circ_SIRT1 has led to attenuation of autophagy and enhancement of response to imatinib in K562/R cells. This circRNA directly binds to the transcription factor EIF4A3 and regulates EIF4A3-mediated transcription of ATG12, thus influencing imatinib resistance and autophagy [79].

In another study, Lu et al. have reported over-expression of circ_0080145, circ_0051886 and ABL1 mRNA, while down-regulation of miR-203 and miR-637 in the non-responder group compared with the responders to imatinib. Notably, ABL1 has been predicted to be a shared direct target of miR-203 and miR-637, two miRNAs that are sponged by circ_0080145 and circ_0051886, respectively. Thus, circ_0080145/miR-203/ABL1 and circ_0051886/miR-637/ABL1 are two molecular axes being involved in imatinib resistance in CML [80]. Table 3 shows the impact of circRNAs on resistance of CML to imatinib.

**Table 3:** Impact of circRNAs on resistance of CML to imatinib (IM).

circRNA	Alternation in CML	Cell line	Database	Patient samples	Mice model	Target	Function	Effects	References
circ_0080145	Upregulation	KCL22/ KG-1/ K562/ KU812/ K562-IM/ KU812-IM	–	peripheral blood specimens from 40 CML patients and 30 healthy donors	nude mice	miR-326/PPFIA1 Axis	Positively regulates PPFIA1 expression through targeting miR-326	Enhances IM resistance	[81]
	Upregulation	K562/K562R	Circinteractome/ miRDB	Blood specimens from 108 CML patients (66 IM-responding and 42 non-responding)	–	miR‑203/ABL1	Promotes the expression of ABL1 mRNA and BCR/ABL1 protein.	Enhances IM resistance	[82]
circCRKL	Downregulation	K562/ K562-G01/KCL22/THP-1/TK6	ENCORI/ circInteractome/ circBank	bone marrow samples of 6 CML patients and 3 healthy controls	NOD/SCID mice	Wnt/β-catenin signaling pathway	Associates with IM sensitivity.	Enhances IM sensitivity	[83]
circ 0051886	Upregulation	K562/k562R	Circinteractome/ miRDB	Blood specimens from 108 CML patients (66 IM-responding and 42 non-responding)	–	miR‑637/ABL1	Promotes the expression of ABL1 mRNA and BCR/ABL1.	Enhances IM resistance	[82]
Circ_0009910	Upregulation	K562 and K562/R	–	65 Serum specimens from CML patients (30 IM-responding and 35 non-responding)	–	MiR-34a-5p/ ULK1	Regulate ULK1-induced autophagy via sponging miR-34a-5p	Enhances IM resistance	[84]
circ_0058493	Upregulation	K562 and K562/G01	Circbank/Circinteractome	Blood specimens from 90 CML patients and plasma samples from 6 IM-responsive and 6 non- responsive patients.	–	miR-548b-3p	Exerts its regulatory function acting as a “sponge” of miR-548b-3p	Enhances IM resistance	[78]

## 2 Discussion

**Fig. 1:**
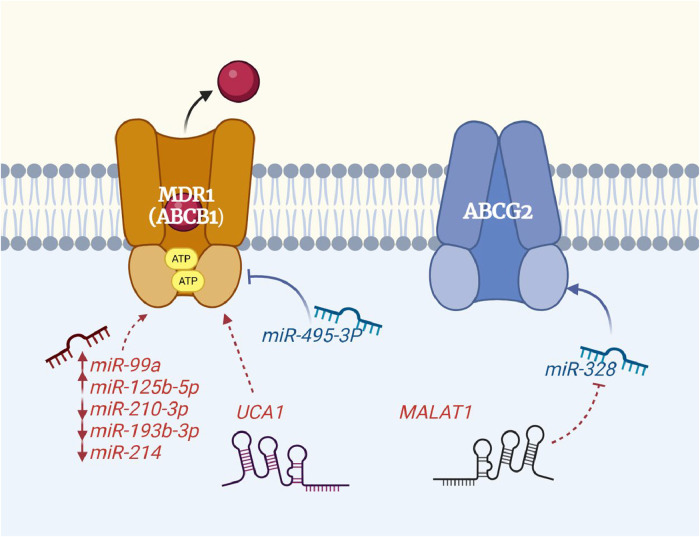
Non-coding RNAs that directly affect BCR-ABL1 protein.

CML is a hematologic malignancy characterized by the presence of the *BCR*-*ABL1* fusion gene, which encodes a constitutively active tyrosine kinase. The introduction of imatinib revolutionized CML treatment, leading to high remission rates. However, a significant proportion of patients develop resistance to imatinib, resulting in disease progression. While BCR-ABL1 kinase domain mutations are a well-known mechanism of resistance, they still leave 20–40 % of resistant cases unexplained [85]. Emerging evidence highlights the critical role of non-coding RNAs in modulating drug sensitivity and resistance. In fact, resistance phenotype is a multifaceted phenomenon that is affected by several cellular/molecular mechanism, namely autophagy, apoptosis, DNA damage response, cancer-related signaling, MDR-related mechanism, alterations in the drug targets or metabolic pathways, disturbance of redox system, and induction of stem cell properties [86]. Theoretically, all of these mechanisms can be involved in the resistance of CML patients to imatinib. While non-coding RNAs can affect almost all steps in resistance mechanism, regulation of expression of MDR proteins seems to be among the most important BCR/ABL1-independent mechanisms of imatinib resistance in CML (Fig. 1).

**Fig. 2:**
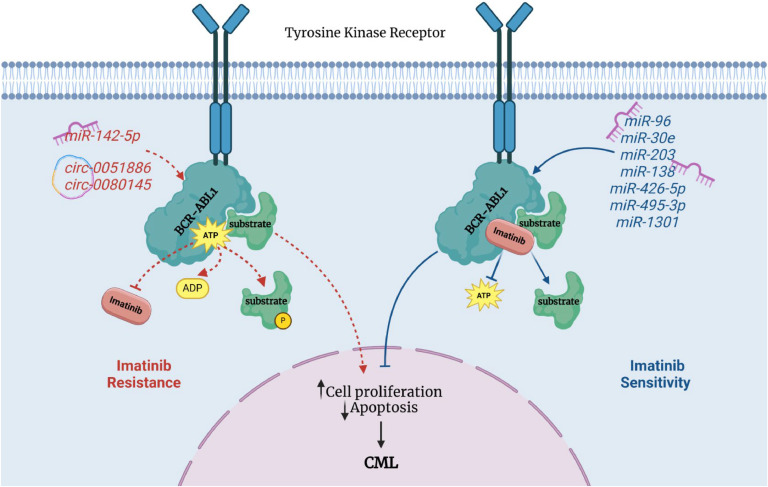
A schematic diagram of the role of miRNAs and circRNAs affecting response to Imatinib through targeting BCR-ABL1. Based on evidences, miR-142-59 [52], circ-0051886 [82] and circ-0080145 [81,82] induce imatinib resistance by dysregulating ABL1 mRNA expression. On the other hand, miR-96 [58], miR-30e [72], miR-203 [30], miR-138 [75], miR-424-5p [70], miR-495–3p [31] and miR-1301 [66] enhance sensitivity to imatinib.

A number of other non-coding RNAs affect BCR-ABL-related mechanisms (Fig. 2).

In fact, identification of molecular mechanisms of imatinib resistance in CML is an active area of research. The advent of genome‐scale screening methods, such as CRISPR knock‐out library has facilitated identification of genes contributing to imatinib resistance. Such studies have shown participation of several genes and pathways, including the Mediator complex, mRNA processing, protein ubiquitinylation, alongside with cancer-related pathways such as MAPK in this process [87]. Thus, non-coding RNAs that regulate these pathways are also putative candidates in this regard.

Several non-coding RNAs have been found to serve as diagnostic biomarkers for imatinib resistance and predictors of clinical outcomes following treatment with this agent. These findings are mainly based on differential expression of non-coding RNAs between resistant and non-resistant samples, including both clinical samples and cell lines. Subsequent *in silico* studies have suggested contribution of a number of signaling pathways in induction of imatinib resistance. These pathways are putative candidates for design of novel therapeutic approaches. Moreover, combination of imatinib with novel strategies that affect expression of specific non-coding RNAs is another way to combat imatinib resistance in CML. These strategies include siRNA- or CRISPR-mediated editing systems.

It is worth mentioning that in addition to BCR-ABL1-related molecules, numerous BCR-ABL1-independent pathways have been found to induce primary resistance to TKIs in CML, particularly though maintenance of leukemic stem cells [88]. Thus, delineating the relation between these pathways and non-coding RNAs is a prerequisite for design of novel therapies. In fact, systematic analysis of lncRNA/miRNA/mRNA and circRNA/miRNA/mRNA networks associated with imatinib resistance would lead to development personalized therapies for CML. Moreover, the detection of actionable mutations—defined as genetic alterations with direct therapeutic implications—complements the study of non-coding RNA-mediated resistance by providing a more inclusive comprehension of treatment failure and managing precision medicine approaches. In fact, these mutations may act in synergy with non-coding RNAs. It is possible that non-coding RNAs modulate the effects of actionable mutations or compensate for their absence. For instance, some miRNAs may influence the expression of mutant BCR-ABL1 or downstream survival pathways. For example, dysregulation of some miRNAs can amplify oncogenic signaling even in the absence of kinase mutations, mimicking a mutation-like phenotype. Additionally, certain lncRNAs can alter chromatin states, promoting resistance by silencing tumor suppressors or activating alternative survival pathways, possibly modulating the effects of certain BCR-ABL1 mutations .Finally, circRNAs may act as mutation buffers, for instance through sponging miRNAs that would otherwise suppress mutant BCR-ABL1 transcripts or compensatory oncogenes.

## 3 Conclusion

Following resistance to imatinib, therapeutic options are limited for CML patients. Thus, there is a vital requisite to find the mechanisms of imatinib resistance. The findings summarized in the current review show that non-coding RNAs have active role in the occurrence of both primary and secondary resistance to imatinib in CML. So, these transcripts are potential targets for management of imatinib resistance in CML. Mechanistically, non-coding RNAs exert their role in imatinib resistance in CML by regulating key signaling pathways. Further research into their mechanisms and clinical applications could lead to innovative strategies for overcoming resistance and refining patient outcomes. Future studies should focus on validating these findings in clinical trials and developing non-coding RNA-based precision therapies.

## Ethics approval and consent to participate

Not applicable.

## Consent for publication

Not applicable.

## Availability of data and material

Not applicable.

## Funding

No funding was received.

## CRediT authorship contribution statement

**Fatemeh Ensafi Talemi:** Conceptualization, Data curation. **Soudeh Ghafouri-Fard:** Writing – original draft, Validation, Writing – review & editing, Supervision.

## Declaration of competing interest

Authors declare no conflict of interests.
